# Voltage-Gated Ca^2+^ Channels in Dopaminergic Substantia Nigra Neurons: Therapeutic Targets for Neuroprotection in Parkinson's Disease?

**DOI:** 10.3389/fnsyn.2021.636103

**Published:** 2021-02-26

**Authors:** Nadine J. Ortner

**Affiliations:** Department of Pharmacology and Toxicology, Institute of Pharmacy, University of Innsbruck, Innsbruck, Austria

**Keywords:** voltage-gated Ca^2+^ channels, Parkinson's disease, Ca^2+^ oscillations, Ca^2+^ channel blockers, L-type Ca^2+^ channels, R-type Ca^2+^ channels, T-type Ca^2+^ channels

## Abstract

The loss of dopamine (DA)-producing neurons in the substantia nigra pars compacta (SN) underlies the core motor symptoms of the progressive movement disorder Parkinson's disease (PD). To date, no treatment to prevent or slow SN DA neurodegeneration exists; thus, the identification of the underlying factors contributing to the high vulnerability of these neurons represents the basis for the development of novel therapies. Disrupted Ca^2+^ homeostasis and mitochondrial dysfunction seem to be key players in the pathophysiology of PD. The autonomous pacemaker activity of SN DA neurons, in combination with low cytosolic Ca^2+^ buffering, leads to large somatodendritic fluctuations of intracellular Ca^2+^ levels that are linked to elevated mitochondrial oxidant stress. L-type voltage-gated Ca^2+^ channels (LTCCs) contribute to these Ca^2+^ oscillations in dendrites, and LTCC inhibition was beneficial in cellular and *in vivo* animal models of PD. However, in a recently completed phase 3 clinical trial, the dihydropyridine (DHP) LTCC inhibitor isradipine failed to slow disease progression in early PD patients, questioning the feasibility of DHPs for PD therapy. Novel evidence also suggests that R- and T-type Ca^2+^ channels (RTCCs and TTCCs, respectively) represent potential PD drug targets. This short review aims to (re)evaluate the therapeutic potential of LTCC, RTCC, and TTCC inhibition in light of novel preclinical and clinical data and the feasibility of available Ca^2+^ channel blockers to modify PD disease progression. I also summarize their cell-specific roles for SN DA neuron function and describe how their gating properties allow activity (and thus their contribution to stressful Ca^2+^ oscillations) during pacemaking.

## Introduction

The primary motor symptoms of the neurodegenerative disorder Parkinson's disease (PD) are caused by a progressive loss of dopamine (DA)-producing neurons in the substantia nigra pars compacta (SN) and associated striatal DA depletion (Obeso et al., 2017). Although PD was first described in 1817 (Parkinson, 2002), to date only symptomatic treatments, but still no cure or disease-modifying therapy, exist (Schulz et al., 2016; Obeso et al., 2017). To develop an effective treatment, it is essential to understand the contributing factors and the disease-underlying cellular mechanisms. Next to globally acting factors (e.g., toxins, aging, and genetic mutations), cell-autonomous ones have also been proposed and widely studied (Poewe et al., 2017; Surmeier et al., 2017b). Neighboring DA neurons in the ventral tegmental area (VTA) share many of the intrinsic properties but are spared in PD (Dauer and Przedborski, 2003). Both are autonomous pacemakers, but in vulnerable SN DA neurons, large oscillations of intracellular Ca^2+^ levels accompany pacemaking (Wilson and Callaway, 2000; Chan et al., 2007; Guzman et al., 2009, 2018; Hage and Khaliq, 2015). In contrast, no or much smaller Ca^2+^ transients were detected in VTA neurons (Guzman et al., 2010, 2018; Benkert et al., 2019) that rely on a different pacemaking mechanism with less contribution of Ca^2+^ currents (Khaliq and Bean, 2010; Philippart et al., 2016). Ca^2+^ influx is important to modulate neuronal excitability and to activate Ca^2+^-dependent physiological processes, but the rhythmic Ca^2+^ load in SN DA neurons also triggers mitochondrial oxidative stress (Guzman et al., 2010, 2018; Surmeier et al., 2017a). Disrupted Ca^2+^ homeostasis and mitochondrial dysfunction are considered key players in PD pathophysiology (Schapira, 2008; Zaichick et al., 2017; Zampese and Surmeier, 2020), and many mutations causing inherited forms of PD affect proteins associated with mitochondrial homeostasis and stress responses (Park et al., 2018). Thus, reducing the activity-related Ca^2+^ load and associated mitochondrial stress in SN DA neurons represents a feasible strategy to increase their resistance to degenerative stressors. L-, R-, and T-type voltage-gated Ca^2+^ channels (LTCCs, RTCCs, and TTCCs, respectively) contribute to the stress-inducing cytosolic Ca^2+^ oscillations, and different approaches to decrease their activity produced promising protective effects in preclinical models of PD. However, a phase 3 clinical trial evaluating the efficacy of LTCC inhibition in early PD patients recently failed. This short review aims to give an overview of the (patho)physiological roles of Ca^2+^ channel activity in SN DA neurons and to (re)evaluate the therapeutic potential of Ca^2+^ channel inhibition (in light of novel preclinical and clinical evidence) and the availability of clinically applicable selective drugs.

## Voltage-Gated Ca^2+^ Channels in SN DA Neurons

**Figure 1:**
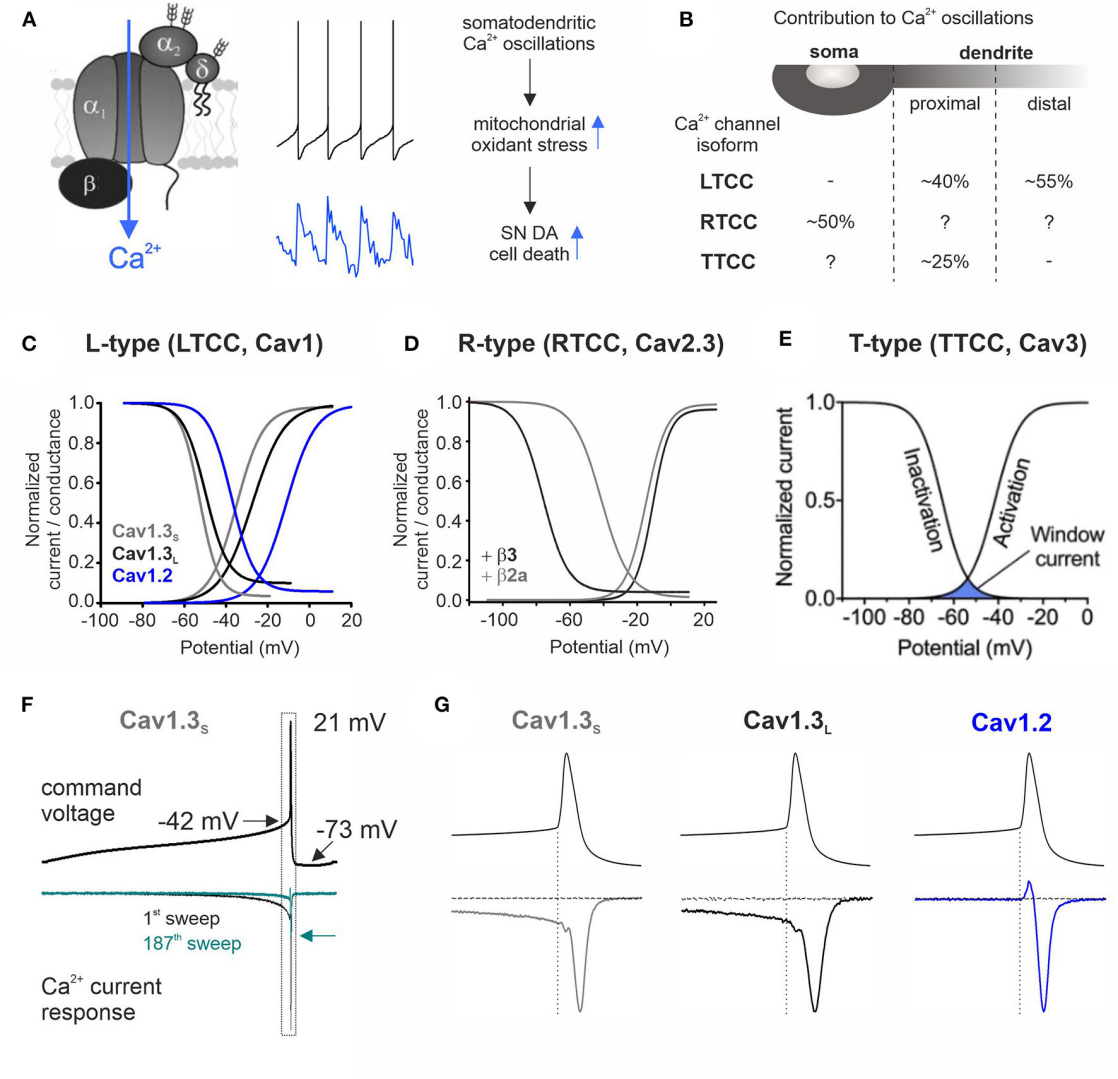
Contribution to stressful somatodendritic Ca^2+^ oscillations, gating properties, and Ca^2+^ current responses of different Ca^2+^ channel isoforms during SN DA neuronal pacemaking. **(A)** Schematic of a plasmalemmal Ca^2+^ channel complex consisting of the pore-forming α1 subunit and auxiliary β and α2δ subunits [only HVA α1 subunits require auxiliary subunits for proper function (Dolphin, 2016)]. Membrane depolarization triggers channel opening and subsequent Ca^2+^ influx. The middle panel shows mouse SN DA neuron pacemaking (top) with the associated intracellular Ca^2+^ transients (bottom; blue) recorded at the cell soma (taken from Ortner et al., 2017). Somatodendritic Ca^2+^ oscillations have been linked to increased mitochondrial oxidative stress, implicated in the high vulnerability of SN DA neurons in PD (Guzman et al., 2010). **(B)** Contribution of different Ca^2+^ channel isoforms to intracellular Ca^2+^ oscillations in distinct cellular compartments (soma and proximal and distal dendrites) (Ortner et al., 2017; Guzman et al., 2018; Benkert et al., 2019). With a higher isradipine concentration [5 μM (Guzman et al., 2018) compared to 1 μM, 10 nM, or chronic *in vivo* isradipine application resulting in plasma levels of ~5 nM isradipine in the abovementioned refs], a complete inhibition of distal dendritic Ca^2+^ transients was observed (Guzman et al., 2009, 2010). Hyphen indicates no contribution found and question mark indicates not determined. **(C–E)** The voltage-conductance (activation) curve describes at which potential a certain Ca^2+^ channel isoform opens (activation threshold) and is in a Ca^2+^ conductive state. The voltage dependence of inactivation gives the proportion of inactivated (non-available) channels at a certain membrane potential. The overlap of these two curves defines the window current (see **E**) that represents the voltage range at which the respective Ca^2+^ channel is steadily active and can thus create a constant background influx of Ca^2+^. **(C)** Cav1.2 or Cav1.3 α1 with β3 and α2δ1 (2 mM Ca^2+^; modified from Ortner et al., 2017). Alternative splicing of the Cav1.3 C-terminus results in functionally distinct long (Cav1.3_L_, black) and short splice variants (Cav1.3s, gray; Singh et al., 2008; Bock et al., 2011; Tan et al., 2011). **(D)** Cav2.3e α1 with β3 (black) or β2a (gray) and α2δ1 (2 mM Ca^2+^). Association with membrane-bound β2a shifts the voltage dependence of inactivation of Cav2.3 channels ~35 mV toward more positive potentials (Olcese et al., 1994; Jones et al., 1998; Yasuda et al., 2004; Miranda-Laferte et al., 2012). **(E)** Voltage dependence of activation and inactivation of TTCCs with indicated window current (blue area; taken from Weiss and Zamponi, 2019a). **(F)** Representative Ca^2+^ current traces (*I*_Ca_, lower panel) through Cav1.3_S_ in response to a murine SN DA neuron action potential command voltage shown above (2 mM Ca^2+^, tsA201 cells; modified from Ortner et al., 2017). Simulated pacemaking (2.5 Hz) resulted in a decrease of *I*_Ca_ to ~20% in steady state (cyan trace, arrow indicates decreased peak *I*_Ca_) through all three investigated Cav1 subtypes (Cav1.2, Cav1.3_S_, and Cav1.3_L_). **(G)** Enlargement of the action potential spike region indicated in **(F)** (dotted rectangle). During the interspike interval (before the spike threshold indicated by the dotted vertical line), only Cav1.3 variants conducted Ca^2+^ while all LTCCs showed Ca^2+^ influx in response to the action potential spike. The horizontal current trace shows full LTCC inhibition at the end of the recording (3 μM isradipine).

Plasmalemmal voltage-gated Ca^2+^ channels mediate controlled Ca^2+^ influx in response to membrane depolarization and contribute to important functions within the sensory, cardiac, endocrine, and nervous systems (Zamponi et al., 2015). Three main families (Cav1/Cav2/Cav3) and 10 individual isoforms are distinguished based on the biophysical and pharmacological properties of the pore-forming α1 subunit: Cav1.1–Cav1.4 (LTCCs), Cav2.1 (P/Q-type), Cav2.2 (N-type), Cav2.3 (RTCCs), and Cav3.1–3.3 (TTCCs) (Catterall, 2011; Zamponi et al., 2015). They are further classified into high-voltage (HVA; Cav1 and Cav2) and low-voltage activated (LVA; Cav3), and only HVA channels require the association with auxiliary β- and α2δ subunits for proper function (Dolphin, 2016; Figure 1A). This functional complexity (further fine-tuned by alternative splicing), their distinct tissue distribution, and subcellular localization enable them to differentially contribute to cellular processes (Dolphin, 2012, 2016; Zamponi et al., 2015).

Rodent SN DA neurons express all voltage-gated Ca^2+^ channel isoforms, except Cav1.1 and Cav1.4 (restricted to skeletal muscle and retina, respectively) (Cardozo and Bean, 1995; Chan et al., 2007; Sinnegger-Brauns et al., 2009; Dufour et al., 2014; Brichta et al., 2015; Shin, 2015; Philippart et al., 2016; Ortner et al., 2017; Guzman et al., 2018; Benkert et al., 2019; Verma and Ravindranath, 2019). The recording of individual current components in intact SN DA neurons is complicated, but in somatic nucleated outside-out patches from juvenile rat SN DA neurons, Ca^2+^ currents of all expressed isoforms were found, with a large LTCC contribution that was higher compared to VTA (Philippart et al., 2016). On the transcript level, RTCC Cav2.3 channels are most abundant in mouse SN DA neurons and levels increase with age (Benkert et al., 2019), while LTCCs (Cav1.2/Cav1.3) get downregulated in an age-dependent manner (Branch et al., 2014; Ortner et al., 2017; Benkert et al., 2019). Of the LVA TTCCs, Cav3.1, and Cav3.2 predominate in mouse SN DA neurons (Poetschke et al., 2015; Guzman et al., 2018; Benkert et al., 2019) and immunohistochemical stainings suggest a rise of somatodendritic TTCCs during development (Dufour et al., 2014) [also shown for Cav1.3, but antibody specificity was not demonstrated in brain (SN) tissue lacking Cav1.3]. SN DA neurons are constantly active and fire action potentials in a tonic single spike or transient high-frequency burst mode *in vivo*, resulting in axonal and somatodendritic DA release (Grace and Bunney, 1984a,b; Chiodo, 1988; Paladini and Roeper, 2014). Cav2 N- and P/Q-type channels drive fast presynaptic neurotransmission, but LTCCs, RTCCs, and TTCCs seem to also contribute to DA release from axonal and/or somatodendritic locations in rodent SN DA neurons (Bergquist and Nissbrandt, 2003; Chen et al., 2006; Brimblecombe et al., 2015; Yee et al., 2019). *In vitro*, even in complete synaptic isolation, SN DA neurons maintain an intrinsically generated regular pacemaker activity (0.5–4 Hz). Inhibition of Na^+^ channels by tetrodotoxin abolished spike generation and revealed slow oscillatory membrane depolarizations (“SOPs,” slow oscillatory potentials; Fujimura and Matsuda, 1989; Yee et al., 2019) that were absent in neighboring VTA neurons and abolished upon LTCC inhibition (Chan et al., 2007). However, LTCCs are not required for pacemaker generation but rather stabilize precision and robustness of pacemaking (Guzman et al., 2009, 2010; Poetschke et al., 2015; Ortner et al., 2017). Similarly, pharmacological inhibition of TTCCs decreased pacemaker precision (juvenile) and frequency (adult mice) (Wolfart and Roeper, 2002; Poetschke et al., 2015), while Cav2.3 knockout or its partial pharmacological inhibition reduced spike amplitude and afterhyperpolarization (AHP) (Benkert et al., 2019). Ca^2+^ influx through voltage-gated Ca^2+^ channels can drive depolarization, but its coupling to K^+^ conductances [e.g., Ca^2+^-sensitive small conductance K^+^ (SK) or A-type K^+^ channels] can also trigger the opposite—a functional coupling important for rhythmic activity (Wolfart and Roeper, 2002; Ji and Shepard, 2006; Duda et al., 2016). During *in vivo* high DA states, Cav1.3 can sensitize the DA-D2 autoreceptor response resulting in activation of G protein-coupled GIRK2 K^+^ channels and inhibition of spiking (Dragicevic et al., 2014). Moreover, voltage-gated Ca^2+^ channels can activate various cellular Ca^2+^-dependent signaling processes, including gene expression as shown for LTCCs (Catterall, 2011; Ma et al., 2012).

## Voltage-Gated Ca^2+^ Channels Contribute to Stressful Somatodendritic Ca^2+^ Oscillations

The slow SN DA neuron pacemaker activity with its broad action potentials triggers large fluctuations of intracellular Ca^2+^ levels throughout the somatodendritic compartment (Chan et al., 2007; Figure 1A, middle panel). Dendritic Ca^2+^ transients occur despite spike inhibition or failure (Chan et al., 2007; Guzman et al., 2018), and their rising phase starts before the action potential threshold (Guzman et al., 2018). This points to a contribution of a Ca^2+^ conductance that is active at subthreshold potentials. Cav1.3 and TTCCs activate well before the spike threshold (as shown for Cav1.3 in tsA201 cells; Ortner et al., 2017; Figure 1G), and their specific gating properties create a “window current” permitting a constant background Ca^2+^ influx through a small fraction of non-inactivated channels (Figures 1C,E) in the average membrane potential range of SN DA neurons (Figure 1F). Pharmacological experiments in mouse brain slices found that LTCCs mediate Ca^2+^ transients in the dendrites (with a higher contribution in distal compartments) (Guzman et al., 2009, 2010, 2018) but not in the soma (Ortner et al., 2017), while TTCCs account for ~25% of proximal dendritic transients (soma not tested) (Guzman et al., 2018; Figure 1B). LTCC dihydropyridine (DHP) inhibitors act on both LTCC isoforms (with higher potency for Cav1.2) (Koschak et al., 2001; Xu and Lipscombe, 2001) and thus cannot be used to discriminate Cav1.2- and Cav1.3-mediated effects. However, using a Cav1.3-specific shRNA-mediated knockdown approach (Guzman et al., 2018), Cav1.3 was identified as the major LTCC isoform underlying the Ca^2+^ oscillations. Similar effects were observed by knockdown and inhibition with DHPs, particularly before spike onset (Guzman et al., 2018) where Cav1.2 is not yet active (Ortner et al., 2017; Figures 1C,G). In the soma, HVA Cav2.3 channels mediate a large proportion of the Ca^2+^ oscillations [~50% in Cav2.3^−/−^ mice; ~25% upon partial pharmacological inhibition by 100 nM SNX-482 (Benkert et al., 2019); dendrites not investigated; Figure 1B]. Cav1.2 and Cav2.3 start to activate at approximately−40 mV (Figures 1C,D) and conduct Ca^2+^ only in response to the strong depolarization of the action potential spike (shown for Cav1.2 in tsA201 cells; Ortner et al., 2017; Figure 1G). In contrast to Cav1.2, Cav2.3 channels typically inactivate at comparatively negative membrane potentials (black trace, steady-state inactivation curve in Figure 1D), which predicts almost complete channel inactivation during SN DA neuron pacemaking. Interaction with membrane-bound β2 splice variants (β2a and β2e) (Buraei and Yang, 2010) was shown to shift Cav2.3 voltage dependence of inactivation toward more positive potentials (Olcese et al., 1994; Jones et al., 1998; Yasuda et al., 2004; Miranda-Laferte et al., 2012, 2014) (gray trace, Figure 1D) and could thereby facilitate Cav2.3 steady-state availability during SN DA neuron activity. β2 accounts for ~30% of β subunits in mouse SN DA neurons (Brichta et al., 2015; Shin, 2015); however, if and to which extent these β2 splice variants are expressed in SN DA neurons and regulate Cav2.3 RTCCs is not known.

Experiments in transgenic mice (mito-GFP mouse) that allow to monitor the oxidation state of mitochondria established a link between Ca^2+^ oscillations and elevated mitochondrial oxidative stress (Guzman et al., 2010, 2018; Figure 1A). Dendritic Ca^2+^ transients and associated mitochondrial oxidation increased with age (Guzman et al., 2018) and were almost absent in neighboring resistant VTA DA neurons (Guzman et al., 2010, 2018; Benkert et al., 2019). In addition, the already high basal oxidant stress level in SN DA neurons was further exacerbated in a genetic PD mouse model (DJ-1 knockout) (Guzman et al., 2010). Interestingly, factors contributing to vulnerability described in SN DA neurons, i.e., slow pacemaking, cytosolic Ca^2+^ oscillations, low intracellular Ca^2+^ buffering (Foehring et al., 2009), and elevated levels of mitochondrial oxidant stress, are also found in other vulnerable non-DA neurons (Surmeier et al., 2017b; Zampese and Surmeier, 2020).

Besides the metabolic challenging pacemaker activity, burst firing is also associated with high Ca^2+^ influx and intracellular Ca^2+^ levels (Hage and Khaliq, 2015; Philippart et al., 2016; Ortner et al., 2017), and membrane hyperpolarizations (e.g., during post-burst pauses) allow channels to recover from inactivation, in particular TTCCs. This mechanism underlies the large TTCC-mediated Ca^2+^ currents and the resulting afterdepolarizations that facilitate rebound spiking (Evans et al., 2017; Tracy et al., 2018). Interestingly, in PD, cells in the ventrolateral part of the SN are particularly prone to cell death and lateral SN DA neurons also show high *in vivo* bursting (Schiemann et al., 2012; Farassat et al., 2019). In line, knockout of K_ATP_ K^+^ channels reduced *in vivo* burst firing and conferred protection in two PD mouse models (Liss et al., 2005; Schiemann et al., 2012).

## Neuroprotection by LTCC Inhibition: Preclinical and Clinical Evidence

Over the last years, LTCCs (and particularly Cav1.3) were considered the main voltage-gated Ca^2+^ channel subtype underlying stressful Ca^2+^ oscillations and thus a major driver of SN DA neuronal cell death (Surmeier et al., 2017a; Guzman et al., 2018; Liss and Striessnig, 2019). Epidemiological studies found that the intake of brain-permeable DHP LTCC inhibitors (antihypertensives) reduced the risk to develop PD (Gudala et al., 2015; Lang et al., 2015; Mullapudi et al., 2016). DHPs have been extensively studied in preclinical PD models and showed promising protective effects in most (but not all) studies (Table 1; reviewed in Leandrou et al., 2019; Liss and Striessnig, 2019). The diverging outcomes of DHP treatment in toxin-based PD animal models have been recently discussed in great detail (Liss and Striessnig, 2019), but no definite explanation has been found. Briefly, in eight out of 13 reports, DHPs significantly reduced mitochondrial-targeting toxin-induced SN DA cell death in mice, rats, and primates (Table 1), but the experimental design of all studies varied, complicating an overall conclusion. Differences included the used PD model (6-OHDA and MPTP), animals (species, strain, age, and sex), treatment regimen (DHP, treatment onset, route of administration, and dosing interval), readout (approach and methodology), and plasma concentrations (if even reported) (Liss and Striessnig, 2019). Thus, a standardized approach in future studies would help to better interpret and compare obtained results. In addition, one recent study (not included in Liss and Striessnig, 2019) tested the DHP felodipine in a genetic PD mouse model (expressing the A53T mutant α-synuclein) and found protective effects on SN DA neuron survival and motor deficits and autophagy-induced clearance of disease-associated proteins from brain (Siddiqi et al., 2019). Noteworthy, in contrast to other DHPs like isradipine (Uchida et al., 1997), felodipine accumulated in the brain with ~2 to 5-fold higher brain levels compared to plasma (Siddiqi et al., 2019). Epidemiological and preclinical evidence, and the availability of safe and clinically approved LTCC DHP inhibitors, prompted the phase 3 STEADY-PD III clinical trial with the DHP isradipine as treatment in early PD patients (336 patients, randomized, double blind, and placebo controlled; Biglan et al., 2017), which however did not reach its primary endpoint (Parkinson Study Group STEADY-PD III Investigators, 2020). Several aspects may have contributed.

**Table 1:** Studies that directly tested neuroprotection by Ca^2+^ channel inhibition, knockdown, or knockout in cellular and animal PD models.

**Model type**	**Drug/intervention**	**Experimental model**	**Outcome/result**	**References**
**LTCCs (Cav1)**				
Cellular	Nifedipine	SH-SY5Ycells (α-synuclein treatment)	Reduction of α-synuclein-induced cell death and Ca^2+^ influx	Melachroinou et al., 2013
	Isradipine	Primary rat ventral midbrain neurons (DA treatment)	Reduction of DA-induced toxicity/cell death and clustering of α-synuclein	Lautenschlager et al., 2018
	Isradipine	Primary mouse midbrain SN DA neurons (MPP^+^ treatment)	Attenuation of MPP^+^-induced toxicity/cell death; mitochondrial oxidation; and intracellular Ca^2+^, DA, and NO elevations	Lieberman et al., 2017
	Nimodipine		Attenuation of MPP^+^-induced toxicity/cell death and intracellular Ca^2+^ and DA elevations	
	Global Cav1.3 knockout		Reduction of cytosolic DA levels compared to WT (untreated and MPP^+^ treated)	
	Nimodipine	Primary mouse ventral midbrain SN DA neurons (L-DOPA treatment)	Reduction of L-DOPA-induced cell loss and cytosolic DA elevation	Mosharov et al., 2009
	Isradipine	PD patient-specific iPSC-derived DA neurons (rotenone treatment)	No rescue of rotenone-induced apoptosis (although a tendency toward a concentration-dependent reduction was observed)	Tabata et al., 2018
	Nifedipine		No rescue of rotenone-induced apoptosis	
Brain slice	Isradipine	Mouse brain slice (rotenone treatment)	Reduction of rotenone-induced SN DA neuron dendritic loss/fragmentation	Chan et al., 2007
	Global Cav1.3 knockout		Reduction of rotenone-induced SN DA neuron dendritic loss/fragmentation	
Animal	Isradipine	PD mouse model (MPTP treatment)	Reduction of MPTP-induced SN DA cell loss and rescue of motor deficits	
	Isradipine	PD mouse model (6-OHDA treatment)	Reduction of 6-OHDA-induced SN DA cell loss	
	Isradipine	PD mouse model (6-OHDA treatment)	Reduction of 6-OHDA-induced SN DA cell loss	Ilijic et al., 2011
	Isradipine	PD mouse model (MPTP treatment)	No rescue of SN DA neuron number or striatal DA content	Price, 2014^*^
	Isradipine	PD mouse model (MPTP treatment)	Reduction of MPTP-induced SN DA cell loss, striatal DA loss, and motor deficits	Wang et al., 2017
	Isradipine	PD mouse model (6-OHDA treatment)	No rescue of SN DA neuron number	Ortner et al., 2017
	Global Cav1.3 knockout		No rescue of SN DA neuron number	
	Nimodipine	PD mouse model (MPTP treatment)	Number of SN DA neurons not determined. No rescue of striatal DA content	Gerlach et al., 1993
	Nimodipine	PD mouse model (MPTP treatment)	Rescue of MPTP-induced SN DA cell loss. No rescue of striatal DA content	Kupsch et al., 1995
	Nimodipine	PD common marmosets model (MPTP treatment)	Rescue of MPTP-induced SN DA cell loss. No rescue of striatal DA content and motor deficits	Kupsch et al., 1996
	Nimodipine	PD rat model (6-OHDA treatment)	No rescue of 6-OHDA-induced SN DA cell loss, striatal DA content and motor deficits	Sautter et al., 1997
	Nimodipine	PD mouse model (MPTP treatment)	Reduction of MPTP-induced SN DA cell loss, striatal DA depletion and motor deficits	Singh et al., 2016
	Nifedipine	PD rat model (6-OHDA treatment)	Number of SN DA neurons not determined. Reduction of 6-OHDA-induced striatal DA depletion and motor deficits	Wang et al., 2012
	Felodipine	Transgenic PD mouse model (SCNA mice expressing the PD-causing A53T mutant α-synuclein)	Reduction of transgene-induced SN DA cell loss, motor deficit and clearance of mutant α-synuclein in mouse brain (via induction of autophagy).	Siddiqi et al., 2019
**RTCCs (Cav2.3)**				
Animal	Global Cav2.3 knockout	PD mouse model (MPTP treatment)	Rescue of MPTP-induced SN DA cell loss	Benkert et al., 2019
**TTCCs (Cav3)**				
Cellular	ML218	PD patient-specific iPSC-derived DA neurons (rotenone treatment)	Reduction of rotenone-induced apoptosis and intracellular Ca^2+^ level elevation	Tabata et al., 2018
	Cav3.1 knockdown		Reduction of rotenone-induced apoptosis and intracellular Ca^2+^ level elevation	
	Cav3.2 knockdown			
	Cav3.3 knockdown			
**Multiple Cav targets**				
Cellular	Benidipine (L + N + T)	PD patient-specific iPSC-derived DA neurons (rotenone treatment)	Reduction of rotenone-induced apoptosis, intracellular Ca^2+^ level elevation, and impaired neurite outgrowth	Tabata et al., 2018

*Summary of studies that evaluated Ca^2+^ channel inhibition, knockdown, or knockout for neuroprotection in PD models. Drugs with multiple mechanisms of action in addition to inhibition of LTCCs, RTCCs, or TTCCs were not included (e.g., zonisamide, cinnarizine, and amiodarone; Tabata et al., 2018). Preclinical studies with LTCC inhibitors have been recently reviewed (Leandrou et al., 2019; Liss and Striessnig, 2019)*.

* *This study has not yet been published in a peer-reviewed journal (only abstract and poster with detailed experimental description). DA, dopamine; iPSC, inducible pluripotent stem cells; MPTP, 1-methyl-4phenyl-1,2,3,6-tetrahydropyridine; MPP^+^, 1-methyl-4-phenyl-pyridine; NO, nitric oxide; SN, substantia nigra; VTA, ventral tegmental area; 6-OHDA, 6-hydroxydopamine*.

Protective DHP effects might require an earlier treatment onset, since at the time when first PD symptoms occur, pathologic mechanisms and neurodegeneration already started—which however would require reliable PD biomarkers (Poewe et al., 2017). In addition, the age-dependent decrease of LTCCs (Branch et al., 2014; Ortner et al., 2017) could limit the therapeutic window of DHPs in PD (especially in elderly patients), although disease state seems to affect LTCC expression [SN neurons from MPTP-treated mice (Verma and Ravindranath, 2019) and post-mortem brains of early-stage PD patients (Hurley et al., 2015) showed robust Cav1.3 levels, despite profound neuron loss]. When considering Cav1.3 as the primary target, the used maximal tolerable isradipine dose [10 mg/day; limited by Cav1.2-mediated peripheral side effects (Parkinson Study Group, 2013)] might be too low to sufficiently engage Cav1.3 LTCCs in SN DA neurons due to their low apparent drug sensitivity (Ortner et al., 2017). This especially applies to C-terminally short splice variants (Huang et al., 2013; Ortner et al., 2017) that are associated with higher Ca^2+^ influx (Singh et al., 2008; Bock et al., 2011; Tan et al., 2011; Figure 1C) and account for ~50% of Cav1.3 transcript in mouse SN DA neurons (Ortner et al., 2017; Verma and Ravindranath, 2019) (higher compared to the cortex and striatum; Verma and Ravindranath, 2019). In this context, the use of an immediate-release isradipine formulation in the STEADY-PD III trial was unfavorable, since slow-onset continuous-release tablets [as used in the phase 2 trial (Parkinson Study Group, 2013), NCT00753636] result in higher average steady-state plasma levels (Liss and Striessnig, 2019). Thus, strategies to increase DHP brain concentrations (Yiu and Knaus, 1996; Ji et al., 2017) or DHPs that accumulate in the brain (e.g., nimodipine and felodipine; Kupsch et al., 1996; Siddiqi et al., 2019) could be an option. Noteworthy, microglia-specific Cav1.2 knockdown augmented MPTP-induced SN DA neurodegeneration and motor deficits in mice, associated with enhanced activation of “neuroinflammatory” M1 microglia (Wang et al., 2019). Thus, Cav1.2 inhibition might even be disadvantageous, which highlights the need for the development of reliable Cav1.3-selective inhibitory drugs. So far, only one putatively selective compound has been described (Cp8 in Kang et al., 2012 and cp-PYT in Cooper et al., 2020) but showed diverging results in follow-up studies from other laboratories (Huang et al., 2014; Ortner et al., 2014). Nevertheless, micromolar concentrations of cp-PYT could lower dendritic transients to a similar extent as isradipine and did not affect Ca^2+^ currents in Cav1.3 knockout mice (Cooper et al., 2020), suggesting Cav1.3-selective inhibition [lack of non-LTCC modulation was also shown in adrenal mouse chromaffin cells (Ortner et al., 2014)]. In the meantime, mice expressing DHP-insensitive Cav1.2 channels could be used to mimic selective pharmacological Cav1.3 inhibition *in vivo* (Sinnegger-Brauns et al., 2004). Lastly, another important aspect is a potential compensation by other Ca^2+^ channel isoforms during chronic isradipine treatment, as found in Cav1.3-deficient mice [upregulation of Cav3.1 TTCCs (Poetschke et al., 2015)]. However, this was not observed with systemic isradipine treatment for 7–10 days in mice [3 μg/g/day; ~5 nM plasma isradipine (Guzman et al., 2018)], but longer exposure has not been tested.

## RTCCs and TTCCs Emerge as Novel PD Drug Targets

Global Cav2.3 knockout fully prevented SN DA neuron degeneration in the gold-standard chronic low-dose MPTP/probenecid PD mouse model (Table 1) and profoundly reduced somatic Ca^2+^ oscillations (~50%, dendrites not tested) (Benkert et al., 2019). Although a direct proof for knockout-induced lowering of high mitochondrial stress levels is missing, a similar inhibition of LTCC-mediated Ca^2+^ transients in proximal and distal dendrites (~35–60%) was sufficient to lower mitochondrial oxidation (Guzman et al., 2010, 2018). The high Cav2.3 levels in vulnerable SN DA neurons (compared to VTA) and their increase with aging further strengthen a possible involvement of RTCCs in PD pathology. Unlike what is observed for Cav1.3 (Poetschke et al., 2015), loss of Cav2.3 did not trigger compensatory upregulation of other Ca^2+^ channels in SN DA neurons (Cav1.2, Cav1.3, and Cav3.1 were tested) (Benkert et al., 2019). In light of these promising findings, it is unfortunate that to date (like for Cav1.3) no selective RTCC inhibitors exist (Schneider et al., 2013). Note that the peptide toxin SNX-482 is selective for Cav2.3 channels only at low concentrations and also inhibits other channels at elevated concentrations (“Cav2.3-prevalent”) (Newcomb et al., 1998; Bourinet et al., 2001; Schneider et al., 2013; Kimm and Bean, 2014). As mentioned above, association of Cav2.3 channels with membrane-bound β2 splice variants (β2a/β2e) may stabilize sustained Cav2.3 activity—a possible explanation for the large contribution of Cav2.3 channels to somatic Ca^2+^ oscillations (~50%, Benkert et al., 2019). Interrupting this putative interaction in SN DA neurons might represent an alternative approach to lower Cav2.3-mediated Ca^2+^ load; however, if these β2 splice variants are indeed expressed in SN DA neurons and modulate Cav2.3 in these cells has not been studied yet. Since β subunits regulate other HVA channels as well, this targeting approach should ideally be Cav2.3-selective [as recently shown for the Cav2.2/β interaction, successfully reducing heterologously expressed and native Cav2.2 currents (Khanna et al., 2019)].

Evidence for a role of TTCCs in PD pathology comes from a study employing DA neurons derived from PD patient-specific iPSCs as *in vitro* PD model (Tabata et al., 2018). Compared to controls, PD patient-specific DA neurons showed pathological signs including reduced neurite length, enhanced oxidative stress, and elevated resting intracellular Ca^2+^ levels and apoptosis. Interestingly, levels of all three TTCC isoforms were also increased. Treatment with the mitochondrial toxin rotenone (“PD trigger”) aggravated the observed effects on cell morphology and survival, which could be prevented by drugs targeting TTCCs as well as individual knockout of all TTCC isoforms. Two of the TTCC inhibitory compounds have complex mechanism of action besides inhibition of TTCCs, but one drug, ML218, specifically blocks TTCCs (Xiang et al., 2011). Interestingly, while LTCC inhibition (nifedipine and isradipine) had no effect, the DHP benidipine that acts on L-, N-, and T-type Ca^2+^ channels could prevent rotenone-induced apoptosis. Although promising, proof of SN DA neuroprotection in *in vivo* PD models is still missing. TTCC inhibition has been explored for the treatment of parkinsonism, but mainly based on their involvement in abnormal burst discharges within the thalamocortical circuitry and associated motor effects (Tai et al., 2011; Kopecky et al., 2014; Yang et al., 2014; Galvan et al., 2016). Zonisamide, an unselective TTCC inhibitor, ameliorated symptoms in PD patients (Murata, 2010), but due to its broad mechanism of action, it is difficult to assign observations to TTCC inhibition. Selective and clinically suitable TTCC inhibitors exist (analgesic/antiepileptic drugs) (Weiss and Zamponi, 2019a,b) and showed good tolerability and safety in phase 2 clinical trials (Richard et al., 2019, 2020), and one phase 2 trial plans to evaluate the selective blocker CX-8998 as treatment for PD-associated tremor (ClinicalTrials.gov #NCT03436953).

## Summary and Conclusion

There is accumulating evidence that voltage-gated Ca^2+^ channels represent an attractive drug target for the therapy of PD. Their contribution to large somatodendritic Ca^2+^ oscillations, associated with increased mitochondrial oxidative stress, seems a likely mechanism by which their activity contributes to SN DA neuron degeneration. Pharmacological and genetic strategies to decrease the activity of LTCCs, RTCCs, and TTCCs showed promising neuroprotective effects in preclinical models of PD, but a phase 3 clinical trial found no slowing of disease progression in early PD patients upon treatment with the DHP LTCC inhibitor isradipine. Although DHPs are safe, brain permeable, and clinically available, their lack of selectivity for Cav1.3, the more likely LTCC target for neuroprotection, and adverse Cav1.2-mediated effects limit their potential for PD therapy. Selective TTCC blockers exist, show good tolerability and safety, and could be repurposed for the therapy of PD; however, evidence for neuroprotection in *in vivo* PD models is still missing. In contrast, no selective inhibitors for Cav1.3 and Cav2.3 exist. The convincing preclinical data described in this review and the discovery of activity-enhancing mutations in neurological diseases (Ortner et al., 2020; Schneider et al., 2020; Weiss and Zamponi, 2020) highlight the urgent need for isoform-selective blockers that are suitable for clinical application. Further studies with available TTCC or multiple Ca^2+^ channel targeting blockers and, if once available, Cav1.3- and Cav2.3-selective inhibitors will help to uncover the full therapeutic potential of Ca^2+^ channel inhibition for neuroprotection in PD.

## Author Contributions

The author confirms being the sole contributor of this work and has approved it for publication.

## Conflict of Interest

The author declares that the research was conducted in the absence of any commercial or financial relationships that could be construed as a potential conflict of interest.
